# Combined occurrence of cardiovascular and bone events in individuals with kidney stone disease

**DOI:** 10.1093/ckj/sfag155

**Published:** 2026-05-18

**Authors:** Pietro Manuel Ferraro, Eric N Taylor, Giovanni Gambaro, Gary C Curhan

**Affiliations:** Section of Nephrology, Department of Medicine, Università degli Studi di Verona, Verona, Italy; Channing Division of Network Medicine, Department of Medicine, Brigham and Women’s Hospital, Harvard Medical School, Boston, MA, USA; Division of Nephrology and Transplantation, Maine Medical Center, Portland, ME, USA; Section of Nephrology, Department of Medicine, Università degli Studi di Verona, Verona, Italy; Channing Division of Network Medicine, Department of Medicine, Brigham and Women’s Hospital, Harvard Medical School, Boston, MA, USA; Division of Renal (Kidney) Medicine, Department of Medicine, Brigham and Women’s Hospital, Harvard Medical School, Boston, MA, USA; Department of Medicine, Harvard Medical School, Boston, MA, USA

**Keywords:** bone health, cardiovascular disease, kidney stone, nephrolithiasis, urolithiasis

## Abstract

**Background:**

Kidney stone disease (KSD) has been linked to increased risk of adverse cardiovascular (CV) and bone events, raising the hypothesis of a shared pathogenic pathway. However, it is unclear whether these events commonly occur in the same stone-forming individual. We examined the occurrence of CV and bone events in individuals with a history of KSD and analyzed their characteristics.

**Methods:**

We analyzed three large prospective cohorts, the Health Professionals Follow-up Study (HPFS, men) and the Nurses’ Health Study (NHS, women) I and II. Individuals with a self-reported diagnosis of KSD during follow-up and no previous CV or bone events at the time of KSD diagnosis were included in the study and followed until the development of a CV event (fatal or non-fatal myocardial infarction, coronary revascularization) and a bone event (fracture, osteoporosis); individuals who developed cancer or died were censored. Characteristics of stone formers experiencing only one event (CV or bone), both events, or none were compared using multinomial logistic regression models.

**Results:**

Our study included data from 14 709 individuals with KSD. In HPFS, 28.7% of the participants experienced at least one event: 17.4% a CV event, 8.5% a bone event and 2.8% both. In NHS I, 46.0% of the participants experienced at least one event: 7.3% a CV event, 32.8% a bone event and 5.9% both. In NHS II, 27.1% of the participants experienced at least one event: 2.0% a CV event, 23.9% a bone event and 1.1% both. Across cohorts, age, BMI, thiazide use, calcium and vitamin D supplementation, and prevalence of hypertension and diabetes did not identify specific high-risk subgroups.

**Conclusion:**

CV and bone events are common in KSD; however, their co-occurrence in the same stone-forming individual is relatively rare. CV disease and bone disease are unlikely to share major risk factors in individuals with KSD.

KEY LEARNING POINTS **What was known:**

- Kidney stone disease (KSD) has been associated with increased risk of both cardiovascular and bone events, suggesting a possible shared pathogenic mechanism, but it was unclear whether these complications commonly co-occur in the same individual with KSD.

**This study adds:**

- In three large prospective cohorts, cardiovascular and bone events were frequent among individuals with KSD, but their co-occurrence in the same person was relatively uncommon. No consistent clinical or metabolic profile identified stone formers at particular risk of developing both complications.

**Potential impact:**

- Findings suggest cardiovascular and bone complications in KSD may arise through distinct pathways, requiring separate risk assessment and management. Future studies should stratify stone patients by outcome risk using tools such as genome sequencing and other “omics” approaches to better guide personalized prevention strategies.

## INTRODUCTION

Kidney stone disease is common, with an estimated prevalence of about 10% [1]. Several extrarenal conditions have been reported to occur more frequently among stone formers, particularly cardiovascular (CV) events [2–5] and reduced bone density and fractures [6–8]. Several hypotheses have been advanced to explain the occurrence of those conditions in individuals affected by kidney stone disease: for instance, since one of the most common and important features of kidney stone formation is excessive urinary excretion of calcium, [9] it was proposed as the potential link between the three conditions, considering that a negative calcium balance induced by excessive loss through urine could also lead to loss of bone mass, although the relationship between urine calcium excretion and CV events would be more difficult to explain. Kidney stone disease, CV disease, and skeletal fragility may share alterations in key regulators of ectopic mineralization, particularly fetuin-A and matrix Gla protein (MGP), as supported by human studies showing in stone formers an association of low fetuin-A with vascular calcification and cortical bone porosity, [10] and MGP polymorphisms associated to both aortic calcification progression and bone loss [11]. A previous study from our group found that stone formers with a history of CVD had lower urinary excretions of citrate and magnesium compared with stone formers without a history of CVD; [12] interestingly, low urine citrate [13, 14] and magnesium [15] are also risk factors for low bone mineral density and fractures.

However, it has never been examined whether both CV and bone events combined occur more within the same individual with kidney stone disease, as one would expect if a common biological link exists between those conditions. In our study, we aim to quantify the proportion of stone formers in whom CV and bone events occur in combination.

## MATERIALS AND METHODS

### Study populations

The Health Professionals Follow-up Study (HPFS) cohort was started in 1986 with the enrollment of 51 529 male health professionals (dentists, optometrists, osteopaths, pharmacists, podiatrists, and veterinarians) aged 40–75 years. The Nurses’ Health Study (NHS) I cohort was started in 1976 with the enrollment of 121 700 female nurses aged 30–55 years. The NHS II cohort was started in 1989 with the enrollment of 116 429 female nurses aged 25–42 years. For each of the cohorts, participants completed a detailed baseline questionnaire with information on lifestyle, medical history and medications. Questionnaires were subsequently mailed every 2 years to update information. This study was approved by the Partners HealthCare Institutional Review Board. Return of completed questionnaires was accepted by the institutional review board as implied informed consent.

### Assessment of kidney stones

Incident kidney stones were defined as self-reported from the participants on the main biennial questionnaire. Self-reported diagnosis was found to be highly reliable by medical record review (confirmed in ≥95% of a sample who completed the supplementary questionnaire) [16]. Stone composition was available for a subgroup of stone formers and was ≥50% calcium oxalate in 77% of NHS I, 79% of NHS II, and 86% of HPFS participants [16].

### Assessment of cardiovascular and bone events

A CV event was considered as a composite of fatal or non-fatal myocardial infarction or coronary revascularization. Fatal myocardial infarction was defined as documented fatal myocardial infarction or fatal coronary heart disease determined by deaths identified from state death certificates or the National Death Index or reported by the participant’s next of kin. Fatal and non-fatal myocardial infarction events were confirmed through medical record review and required characteristic symptoms with either diagnostic electrocardiographic changes or positive myocardial enzymes; revascularization was self-reported but has previously been found to be virtually 100% specific in the HPFS cohort [17].

A bone event was considered as a composite of a self-reported fracture (vertebral or hip), incident osteoporosis or incident osteopenia. Self-reported low bone mass and fractures were previously validated using medical records, and found to be valid [18, 19].

### Assessment of covariates

Baseline information including body mass index, history of hypertension, history of diabetes, use of thiazides and bisphosphonates was obtained from the main biennial questionnaire. Use of calcium and vitamin D supplements was obtained from the standardized food frequency questionnaire obtained every 4 years.

### Statistical analysis

First, we excluded participants who had either a history of kidney stones (to reduce the likelihood of previous CV/bone events occurring before study enrollment not being recorded) or a previous CV or bone event at baseline. Individuals who developed a kidney stone during follow-up were then included in the analysis and followed until they developed a CV event, a bone event, non-CV death or loss to follow-up. If a participant experienced either a CV or a bone event during follow-up, they would remain in the risk set until they developed any of the other outcomes. Participants were then categorized into “No events” (those who did not experience either a CV nor a bone event), “CV events only,” “Bone events only,” and “Combined events,” and their baseline characteristics were compared across groups with cohort-specific multinomial logistic regression models using the outcome event as the dependent variable and baseline age, BMI, use of thiazides, use of calcium supplements, use of vitamin D supplements, history of hypertension, and history of diabetes as independent variables. Results of the multinomial regression models were pooled across cohorts using random-effects meta-analysis. All analyses were repeated after censoring individuals who developed fatal myocardial infarction as their first event, since it could be considered as a competing event for the development of a bone event.

A two-tailed *P*-value < .05 was considered statistically significant. All analyses were performed with SAS version 9.4.

## RESULTS

**Figure 1:**
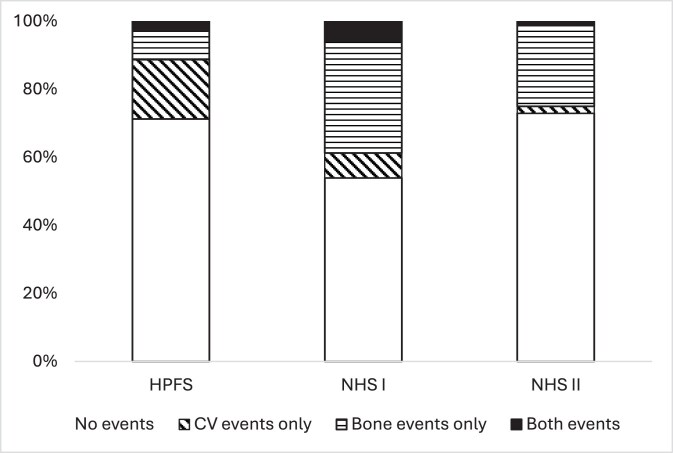
Proportion of participants experiencing CV and/or bone events during follow-up. HPFS, Health Professionals Follow-up Study; NHS, Nurses’ Health Study; CV, cardiovascular.

The study included data from 14 709 individuals who developed a first kidney stone during follow-up; their baseline characteristics according to the type of event experienced are reported in Table 1. The proportion of participants in each cohort developing a CV and/or bone event is reported in Fig. 1. In HPFS, 28.7% of the participants experienced at least one event: 17.4% a CV event, 8.5% a bone event and 2.8% both events. In NHS I, 46.0% of the participants experienced at least one event: 7.3% a CV event, 32.8% a bone event, and 5.9% both events. In NHS II, 27.1% of the participants experienced at least one event: 2.0% a CV event, 23.9% a bone event, and 1.1% both events. Thus, in general, events of interest were more common in NHS I compared with the other cohorts; CV events were more common among the male HPFS participants, and bone events among the female NHS participants.

**Table 1:** Baseline characteristics by cohort and event groups.

	HPFS			
	No events (*n* = 2893)	CV events only (*n* = 708)	Bone events only (*n* = 343)	Combined events (*n* = 115)
Age (years)	62.5	60.3	61.3	60.0
BMI (kg/m^2^)	26.5	26.9	25.8	26.0
Use of thiazides (%)	7.1	6.8	3.8	5.2
Use of calcium supplements (%)	20.1	21.9	22.2	15.9
Use of vitamin D supplements (%)	11.9	5.8	6.0	1.0
High blood pressure (%)	41.8	44.4	30.3	46.1
Diabetes (%)	8.5	10.6	5.5	7.8
	NHS I			
	No events (*n* = 2128)	CV events only (*n* = 289)	Bone events only (*n* = 1294)	Combined events (*n* = 233)
Age (years)	62.0	59.0	57.6	58.0
BMI (kg/m^2^)	28.1	29.3	26.5	27.5
Use of thiazides (%)	12.5	15.0	9.9	17.5
Use of calcium supplements (%)	43.5	34.5	38.0	32.4
Use of vitamin D supplements (%)	21.7	6.6	10.4	9.3
High blood pressure (%)	52.5	62.8	37.6	52.2
Diabetes (%)	13.8	24.9	8.0	15.1
	NHS II			
	No events (*n* = 4892)	CV events only (*n* = 136)	Bone events only (*n* = 1605)	Combined events (*n* = 73)
Age (years)	48.6	48.8	46.3	45.7
BMI (kg/m^2^)	29.4	32.1	27.1	29.9
Use of thiazides (%)	6.3	11.6	5.3	12.9
Use of calcium supplements (%)	32.8	33.6	34.6	34.4
Use of vitamin D supplements (%)	20.7	18.8	12.7	8.5
High blood pressure (%)	33.8	48.6	26.1	48.6
Diabetes (%)	9.9	29.0	6.9	22.9

BMI, body mass index; HPFS, Health Professionals Follow-up Study; NHS, Nurses’ Health Study.

The results of the multinomial logistic regression models are reported in Table 2. In summary, when using no events as the referent group (top part of Table 2), age was significantly associated with lower odds of any outcome, BMI was positively associated with CV events and inversely with bone events and combined outcomes, use of calcium supplements was positively associated with bone events, use of vitamin D supplements was inversely associated with each outcome, and high blood pressure and diabetes were positively associated with CV and combined events. Changing the referent category to combined events, BMI and diabetes were directly associated with higher odds of CV events, whereas age (directly), high blood pressure, and diabetes (inversely) were associated with higher odds of bone events.

**Table 2:** Odds ratios from multinomial logistic regression models for different event groups.

	CV events only	Bone events only	Combined events
	Ref = “No events”		
Age (per year)	0.98 (0.97, 0.98)*P* < .001	0.98 (0.97, 1.00)*P = .01	0.97 (0.95, 0.98)*P* < .001
BMI (per kg/m^2^)	1.01 (1.00, 1.03)*P* = .046	0.96 (0.95, 0.97)*P* < .001	0.98 (0.96, 1.00)*P* = .04
Use of thiazides	1.00 (0.79, 1.26)*P* > .99	1.01 (0.85, 1.20)*P* = .92	1.21 (0.79, 1.85)*P* = .37
Use of calcium supplements	1.18 (0.97, 1.42)*P* = .09	1.27 (1.15, 1.40)*P* < .001	1.08 (0.85, 1.37)*P* = .52
Use of vitamin D supplements	0.46 (0.28, 0.74)*P* = .001	0.56 (0.48, 0.65)*P* < .001	0.38 (0.20, 0.74)*P* = .003
High blood pressure	1.41 (1.12, 1.77)*P* = .003	0.84 (0.69, 1.03)*P = .09	1.46 (1.15, 1.86)*P* = .002
Diabetes	2.16 (1.37, 3.43)*P = .001	0.92 (0.74, 1.14)*P* = .45	1.59 (0.97, 2.61)*P* = .07
	Ref = ‘CV events only’		
Age (per year)	–	1.01 (1.00, 1.02)*P* = 0.027	1.00 (0.98, 1.01)*P* = 0.53
BMI (per kg/m^2^)	–	0.95 (0.94, 0.97)*P* < .001	0.97 (0.95, 0.99)*P* = .007
Use of thiazides	–	0.90 (0.67, 1.21)*P* = .48	1.14 (0.77, 1.71)*P* = .51
Use of calcium supplements	–	1.10 (0.90, 1.35)*P* = .35	0.98 (0.73, 1.31)*P* = .88
Use of vitamin D supplements	–	1.18 (0.68, 2.06)*P* = .56	0.72 (0.20, 2.52)*P = .61
High blood pressure	–	0.56 (0.44, 0.70)*P* < .001	1.05 (0.69, 1.61)*P* = .81
Diabetes	–	0.39 (0.29, 0.54)*P* < .001	0.68 (0.47, 0.98)*P* = .038
	Ref = “Bone events only”		
Age (per year)	0.99 (0.98, 1.00)*P* = .027	–	0.98 (0.97, 1.00)*P* = .04
BMI (per kg/m^2^)	1.05 (1.04, 1.07)*P* < .001	–	1.02 (1.00, 1.04)*P* = .09
Use of thiazides	1.11 (0.83, 1.50)*P* = .48	–	1.30 (0.92, 1.84)*P* = .14
Use of calcium supplements	0.91 (0.74, 1.11)*P* = .35	–	0.88 (0.69, 1.13)*P* = .32
Use of vitamin D supplements	0.85 (0.49, 1.47)*P* = .56	–	0.72 (0.41, 1.29)*P* = .27
High blood pressure	1.80 (1.43, 2.26)*P* < 0.001	–	1.81 (1.42, 2.31)*P* < 0.001
Diabetes	2.55 (1.86, 3.50)*P* < .001	–	1.78 (1.27, 2.51)*P* < .001
	Ref = “Combined events”		
Age (per year)	1.00 (0.99, 1.02)*P* = .53	1.02 (1.00, 1.03)*P* = .04	–
BMI (per kg/m^2^)	1.03 (1.01, 1.06)*P* = .007	0.98 (0.96, 1.00)*P* = .09	–
Use of thiazides	0.87 (0.59, 1.30)*P* = .51	0.77 (0.54, 1.09)*P* = .14	–
Use of calcium supplements	1.02 (0.76, 1.37)*P* = 0.88	1.14 (0.89, 1.46)*P* = 0.32	–
Use of vitamin D supplements	1.39 (0.40, 4.90)*P = .61	1.38 (0.78, 2.46)*P* = .27	–
High blood pressure	0.95 (0.62, 1.46)*P* = .81	0.55 (0.43, 0.71)*P* < .001	–
Diabetes	1.47 (1.02, 2.11)*P* = .04	0.56 (0.40, 0.79)*P* = .001	–

Results are reported as odds ratios and 95% confidence intervals. All models simultaneously include all independent variables. BMI, body mass index.

*Statistically significant heterogeneity across cohorts.

## DISCUSSION

Over time, several studies have reported that individuals with kidney stone disease have a higher proportion of bone abnormalities including osteoporosis and osteopenia, abnormal bone microarchitecture and even a higher risk of incident fractures [6, 7, 20]. Several explanations for this phenomenon have been proposed, including negative calcium balance due to abnormal tubular calcium handling, dysregulation of 1,25-dihydroxyvitamin D physiology, and excessive dietary salt and animal protein, which are also risk factors for development of bone disease [21, 22].

More recently, a link between kidney stone disease and CV outcomes has been established from observational studies, [2–4] a finding confirmed by studies employing a Mendelian randomization approach [23]. However, in a highly heterogeneous condition such as kidney stone disease, to hypothesize a shared pathogenic pathway between CV and bone derangements requires those to be observed in the same individual, otherwise it could be hypothesized that certain stone formers are more prone to CV complications and others to bone complications. Our data, showing a relatively low proportion of incident stone formers developing both outcomes (ranging from 1.1% in NHS II to 5.9% in NHS I), would suggest that the circulatory system and the bone are affected by different derangements in the stone forming population. Alternatively, it is possible that the occurrence of one outcome would preclude the subsequent occurrence of the other; this could be especially true for potentially fatal CV events, but even fractures are a known risk factor for subsequent mortality [24]. This possibility is further suggested by the relatively lower prevalence of diabetes and high blood pressure, two major risk factors for CV outcomes, in those participants who developed only bone events. We tried to account for this phenomenon by censoring individuals developing a fatal CV event, but our findings remained almost unchanged.

In our study, we found several variables that were statistically significantly associated with developing either one outcome or both, compared to none; however, the magnitudes of the associations were generally modest and unlikely to explain systematic differences. It is more likely that for several variables the statistical significance is explained by the large sample size of the study. Future studies will need to probe further by characterizing stone patients according to their likelihood to develop certain outcomes over time, such as genome sequencing and other “omics” approaches.

Our study has several strengths, including its longitudinal prospective design allowing to establish a clear temporal line between the exposure and the outcomes of interest, the large sample size of incident stone formers and the use of validated clinical information. Our study also has some limitations. For instance, the majority of the participants were white, and we did not have information available on urine chemistries, stone composition or kidney function for most of the cohort population.

In conclusion, the simultaneous occurrence of CV and bone events in stone patients is relatively rare. The association between stones and these other conditions are likely due to distinct mechanisms.

## Data Availability

All data generated or analyzed during this study are included in this published article; further enquiries can be directed to the corresponding author.
